# ROBOKOP: an abstraction layer and user interface for knowledge graphs to support question answering

**DOI:** 10.1093/bioinformatics/btz604

**Published:** 2019-08-13

**Authors:** Kenneth Morton, Patrick Wang, Chris Bizon, Steven Cox, James Balhoff, Yaphet Kebede, Karamarie Fecho, Alexander Tropsha

**Affiliations:** 1 CoVar Applied Technologies, Durham, NC, USA; 2 Renaissance Computing Institute; 3 School of Pharmacy, University of North Carolina at Chapel Hill, Chapel Hill, NC, USA

## Abstract

**Summary:**

Knowledge graphs (KGs) are quickly becoming a common-place tool for storing relationships between entities from which higher-level reasoning can be conducted. KGs are typically stored in a graph-database format, and graph-database queries can be used to answer questions of interest that have been posed by users such as biomedical researchers. For simple queries, the inclusion of direct connections in the KG and the storage and analysis of query results are straightforward; however, for complex queries, these capabilities become exponentially more challenging with each increase in complexity of the query. For instance, one relatively complex query can yield a KG with hundreds of thousands of query results. Thus, the ability to efficiently query, store, rank and explore sub-graphs of a complex KG represents a major challenge to any effort designed to exploit the use of KGs for applications in biomedical research and other domains. We present Reasoning Over Biomedical Objects linked in Knowledge Oriented Pathways as an abstraction layer and user interface to more easily query KGs and store, rank and explore query results.

**Availability and implementation:**

An instance of the ROBOKOP UI for exploration of the ROBOKOP Knowledge Graph can be found at http://robokop.renci.org. The ROBOKOP Knowledge Graph can be accessed at http://robokopkg.renci.org. Code and instructions for building and deploying ROBOKOP are available under the MIT open software license from https://github.com/NCATS-Gamma/robokop.

**Supplementary information:**

Supplementary data are available at *Bioinformatics* online.

## 1 Introduction

A knowledge graph (KG) uses an appropriate ontology to express domain knowledge as a graph of relationships (edges) between entities (nodes), with related nodes connected by edges. KG databases such as Neo4j allow KGs to be queried using a query language such as Cypher that is designed to find matching relationship paths or sub-graphs within the KG. Queries intended to find longer paths or larger sub-graphs often result in an explosion in the number of matching paths. To facilitate rapid exploration of a KG for hypothesis generation or exploration of identified relationships, an interface is required to enable a user to quickly explore the results of complex queries without the need to write custom commands or computer code. Although tools exist for visually querying and exploring the results of a KG such as the Neo4j browser, these tools are often insufficient for queries that return numerous paths that are best represented as sub-graphs.

Herein, we describe Reasoning Over Biomedical Objects linked in Knowledge Oriented Pathways (ROBOKOP) and focus on capabilities enabled by the ROBOKOP user interface (UI). ROBOKOP was motivated by our work on the Biomedical Data Translator program (‘Translator’), which is funded by the National Center for Advancing Translational Sciences (The Biomedical Data Translator Consortium, 2019a, b). ROBOKOP is comprised of a biomedical ROBOKOP KG that is stored within a Neo4j database and queried using the Cypher query language (hosted at http://robokopkg.renci.org).

KGs of biomedical concepts and associate software to visualize and explore them have had several recent implementations such as BioGraph (Liekens *et al.*, 2011) and Het.io (Himmelstein *et al.*, 2017). ROBOKOP provides a unique query mechanism based on meta-graphs (Huang *et al.*, 2016) and a novel ranking algorithm. The ROBOKOP software stack includes a web server, application programming interface (API) and a web-based UI that together enable users to create queries in an easy-to-use format, store the results of those queries, rank the relevance of the queries and graphically explore the results. Using ROBOKOP and the underlying database, users can explore connections between biomedical entities to answer directed questions such as ‘*what genes are associated with Ebola?*’ or explore more complex paths such as ‘*find a clinical outcome pathway that provides a mechanistic explanation for the effectiveness of imatinib in the treatment of asthma*’.

## 2 Implementation

### 2.1 Query specification

ROBOKOP queries are specified using a JSON-based template for the requested sub-graph. The query specification is a meta-graph (e.g. Fang *et al.*, 2016; Huang *et al.*, 2016; Zhao *et al.*, 2017), which is a generalization of meta-paths (e.g. Cao *et al.*, 2017; Sun *et al.*, 2011) that incorporates general graph structure. Each node in the query sub-graph represents an entity specified by a type, with edges between nodes representing a specified relationship between the entities. Each node can be further specified by additional properties, including identifiers or other meta-data. Edge types can be specified to limit the allowable relationships between entities; if multiple edge types are given, then edges that match any of the specified types will be returned. Matches to the query, known as answers, are sub-graphs that match the template in topology and the types and desired properties of the nodes and edges. Within the query, each template node and edge must be assigned a unique identifier that is used to bind the results of the query. Of note, this meta-graph–based query specification evolved into the Translator API standard specification (The Biomedical Data Translator Consortium, 2019b).

### 2.2 Query answers

ROBOKOP answers are stored using the identifiers of the nodes and edges in the query specification as bindings and the identifiers in the local KG as references. This format is more compact than naïve storage approaches, as nodes and edges that are used in multiple answers do not require complete meta-data for duplication. In addition, the nodes and edges within each answer are bound to the nodes and edges of the query specification; this makes exploration of complex queries containing repeated nodes types, or even repeats of the same node, transparent.

### 2.3 Answer-ranking algorithm

Queries that are generated with little specification regarding nodes and edges or with multiple nodes and edges typically result in numerous matching sub-graphs. As such, the rank of sub-graphs by relevance to the query and strength of the supporting evidence is critical for user exploration of results. The ROBOKOP answer-ranking algorithm weights each edge within each sub-graph using a metric that is based on the number of PubMed abstracts that cite both the source and target nodes. The publication support is provided by an additional ROBOKOP service, termed OmniCorp, that contains a graph of PubMed identifiers linked to concepts (i.e. potential ROBOKOP KG nodes) referenced within abstracts. OmniCorp is built by processing all PubMed abstracts with the SciGraph Named Entity Recognition API (https://github.com/SciGraph/SciGraph/) and matching text in titles and abstracts to concepts from a predetermined set of biomedical ontologies. A confidence score for each answer is calculated based on the resistance distance (Klein and Randić, 1993) between leaves of the answer sub-graph, using weights derived from the publication counts provided by curated data sources and publication co-occurrence counts provided by OmniCorp, with the former treated with greater importance than the latter.

### 2.4 API and UI

The ROBOKOP web-based UI enables users to specify queries, store and recall query results and graphically explore the ranked list of answers. The storage format for results allows users to filter answers by the unique instances assigned to each node in the query specification. This approach enables users to quickly explore thousands of potential sub-graphs, even when the ranking of answers suggests that a given sub-graph may not be relevant to a specific query. For instance, certain users may be less interested in identifying answers with a substantial amount of existing publication support than in identifying answers that may have little publication support, but might suggest new insights or hypotheses for subsequent testing. The metadata for each node and edge, including provenance information, can be viewed, as well as relevant supporting publications. A series of publicly accessible APIs are used by the UI to communicate with server-side software (http://robokop.renci.org/apidocs).

## 3 Conclusion

The ROBOKOP backend and UI can be used to explore the ROBOKOP KG and identify answers to biomedical queries or generate hypotheses for future biomedical research. Questions and ranked answers are stored and can be explored in a customized ROBOKOP UI. The ROBOKOP query specification and query format enable efficient storage of graph-structured answers.

ROBOKOP is under active development, with performance and feature enhancements deployed regularly. Planned development includes additional methods for local KG exploration, techniques for more rapid query iteration and integration with graph-based machine-learning methods.

A public instance of the KG is available at http://robokopkg.renci.org; the UI is available at http://robokop.renci.org/ and an API is available at http://robokop.renci.org/apidocs.

## Supplementary Material

btz604_Supplementary_Data
