# Supplementary material for: ROBOKOP: an abstraction layer and user interface for knowledge graphs to support question answering
Source: Bioinformatics. 2019 Aug 13;35(24):5382–4. doi: 10.1093/bioinformatics/btz604 (PMC6954664; doi:10.1093/bioinformatics/btz604)
Supplement: btz604_Supplementary_Data [file btz604_supplementary_data.zip › btz604-Suppl_data/Supplementary_Data2.docx]

**Supplementary Information**

ROBOKOP: An Abstraction Layer and User Interface for Knowledge Graphs to Support Question Answering

Kenneth Morton^1,*^, Patrick Wang^1^, Chris Bizon^2^, Steven Cox^2^, James Balhoff^2^, Yaphet Kebede^2^,

Karamarie Fecho^2^, Alexander Tropsha^3^

^1^CoVar Applied Technologies, Durham, North Carolina, USA, ^2^Renaissance Computing Institute, University of North Carolina at Chapel Hill, Chapel Hill, North Carolina, USA; ^3^School of Pharmacy, University of North Carolina at Chapel Hill, Chapel Hill, North Carolina, USA

*To whom correspondence should be addressed.

# ROBOKOP

ROBOKOP (Reasoning Over Biomedical Objects linked in Knowledge Oriented Pathways) is a question-answering system that is built upon multiple open biomedical databases and designed to explore relationships between a variety of biomedical data types. With ROBOKOP, varied biomedical data are semantically integrated into a Knowledge Graph (KG) that provides access to high-level concepts connected by Knowledge Sources. Nodes in the graph represent entities such as chemical substance, gene, biological pathway or process, cell type, anatomical structure or system, and disease. Edges between nodes provide predicates that explain the association between the connected nodes; the predicates include concepts such as “treats”, “causes”, or “is associated with”. Edges also provide publication support for connections between nodes, i.e., co-occurrence of terms in PubMed abstracts. Using a structured query language, users posit questions, such as: “*Why does imatinib help people with asthma?” “Why does clonidine interact with propranolol?” “Why is sickle cell disease protective against malaria?”* ROBOKOP then locates applicable answer subgraphs in its KG and scores these putative answers, allowing users to view the results and supporting evidence (i.e., PubMed literature citations) via the ROBOKOP user interface (UI).

The ROBOKOP KG contains approximately 500,000 nodes (entities) and 12M edges (relationships). The open ROBOKOP UI can be accessed at:<http://robokop.renci.org>; a publicly available version of the database can be downloaded at [http://robokopkg.renci.org](http://robokopkg.renci.org/). A simplified version of the ROBOKOP KG schema is shown in Figure 1. An edge connecting two entities means that one or more data sources connecting entities of those types has been integrated into the ROBOKOP KG.


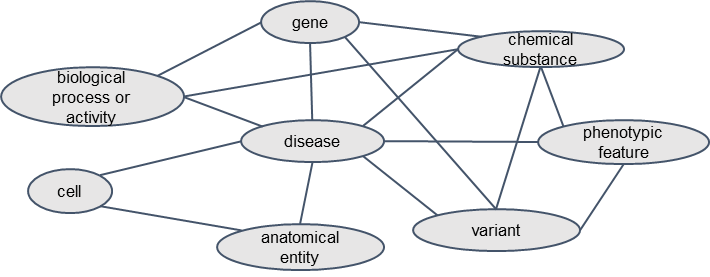


**Fig. 1. Simplified schema for ROBOKOP KG.**

# Multiplex Queries (Sets)

ROBOKOP queries can allow multiple entities to simultaneously occupy the same role in the response. For instance, consider a query requesting diseases that present symptoms or phenotypes similar to those of Fanconi anemia. We could represent that query as:

*Fanconi anemia -> *phenotypic_feature -> disease*,

where the star indicates that the phenotypic features can be multiplexed. Each answer to this question includes a single disease along with a variable number of phenotypic features. For example, one of the highest-scoring results identifies the disease aplastic anemia, which shares 335 phenotypic features with Fanconi anemia.

This query functionality allows users to explore entity relationships/interactions that are mediated by multiple biological mechanisms. The ability to compare the graphs representing such relationships was one motivation for ROBOKOP’s resistance distance–based answer-scoring mechanism.

# ROBOKOP Queries for COPS

ROBOKOP can respond to arbitrary graph queries across biomedical topics and is being used to explore a variety of clinical outcome pathways (COPs) and adverse outcome pathways (AOPs). For example, ROBOKOP was used to generate a COP-type question designed to investigate the mechanism of action of nifedipine in the treatment of cardiovascular disease. This question was posed to ROBOKOP via the ROBOKOP UI as a graph query finding a linear path starting at nifedipine and ending at cardiovascular disease, and traversing a specified set of node types in between:

*nifedipine -> gene -> biological process or activity -> phenotypic feature -> cardiovascular disease*.

Users can pose and view the question, as well as the output list of ranked answers, in the ROBOKOP UI. As seen in the screenshot in Figure 2, multiple answers, representing different paths through the KG are returned. These potential answers are scored and ranked through an algorithm that factors in the number of supporting PubMed publications, as well as indirect support provided by literature co-occurrence of all pairwise sets of terms in the path.


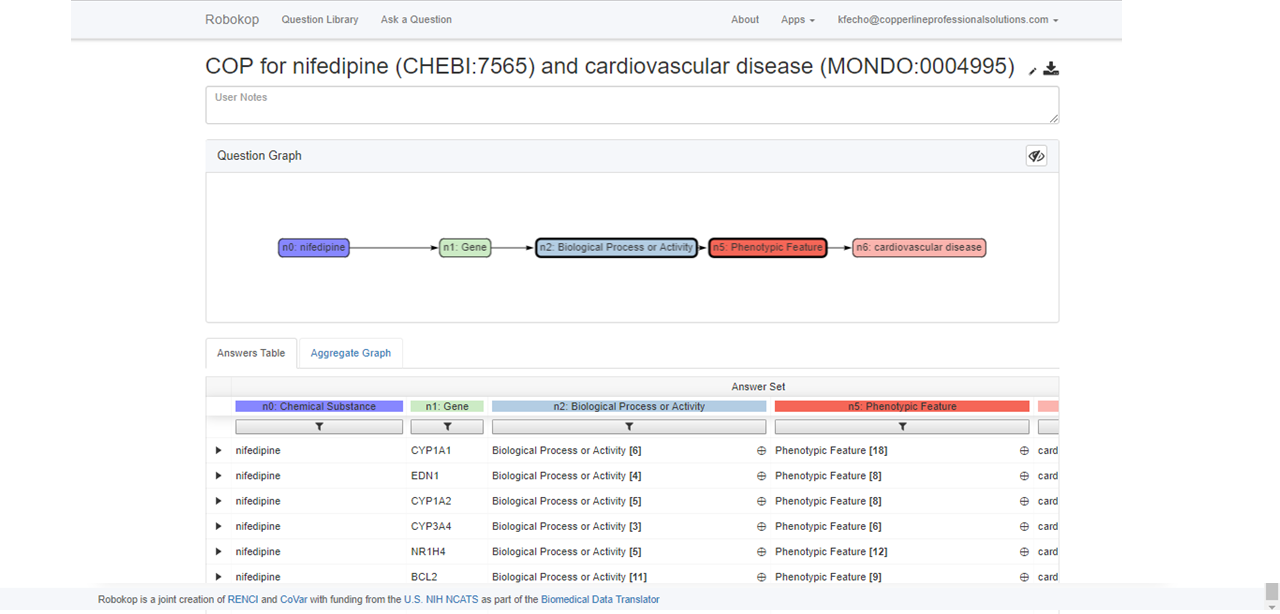


**Fig. 2. Example COP query.**

The top answer for this COP is shown in Figure 3.


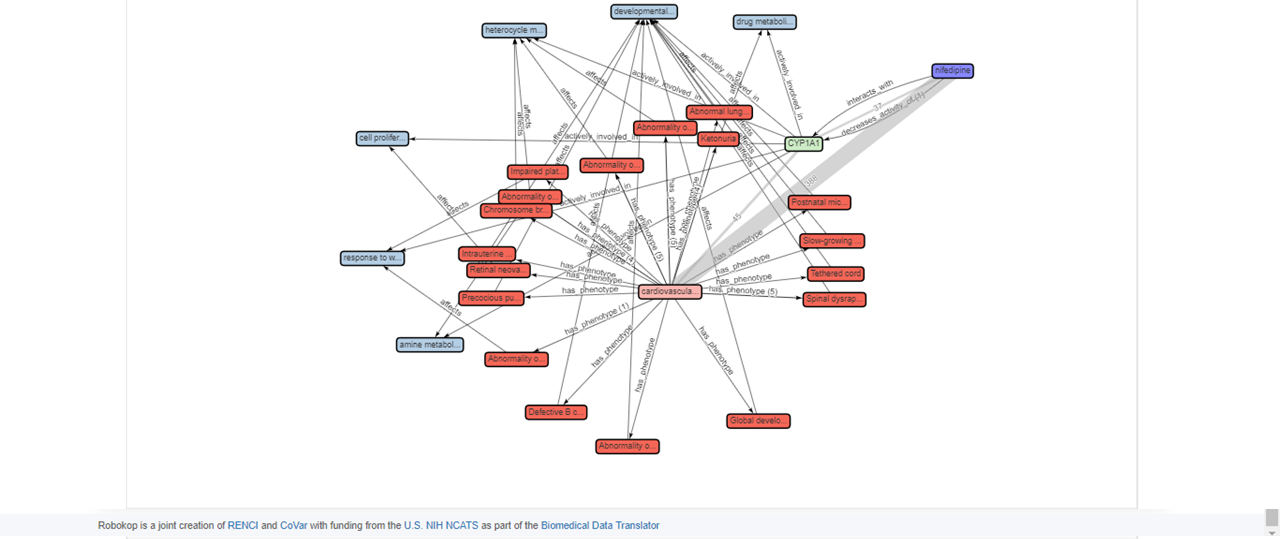


**Fig. 3. Example answer subgraph for COP query.**

Users can interact with the graph and explore the supporting publications. In this case, ROBOKOP identified 37 PubMed articles as supporting evidence for the *nifedipine* -> *CYP1A* relationship in the first answer subgraph. (One additional article was provided from the underlying data source, in this case the Comparative Toxicogenomics Database [CTD].) Users can view the citations (the first one is shown in Figure 4) and click on the button to the left of the reference to retrieve the full PubMed abstract.


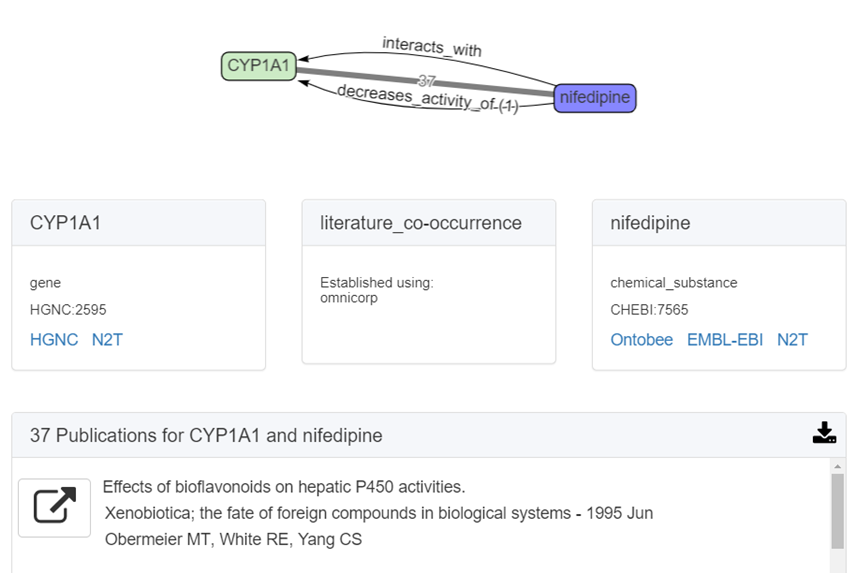


**Fig. 4. Example supporting evidence for COP query answer.**

# ROBOKOP Queries for AOPS

**
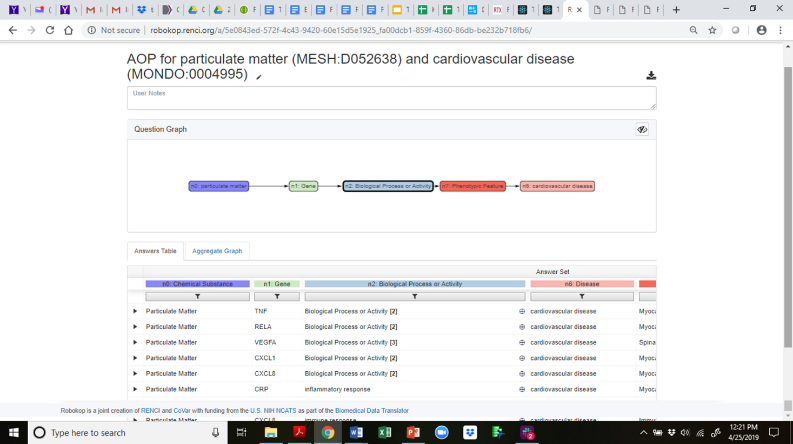
**Similarly, ROBOKOP can be used to generate AOPs. The example shown in Figure 5 shows an AOP-type question that was posed to ROBOKOP via the ROBOKOP UI and intended to explore the negative effects of exposure to particulate matter on cardiovascular disease.

**Fig. 5. Example AOP query.**


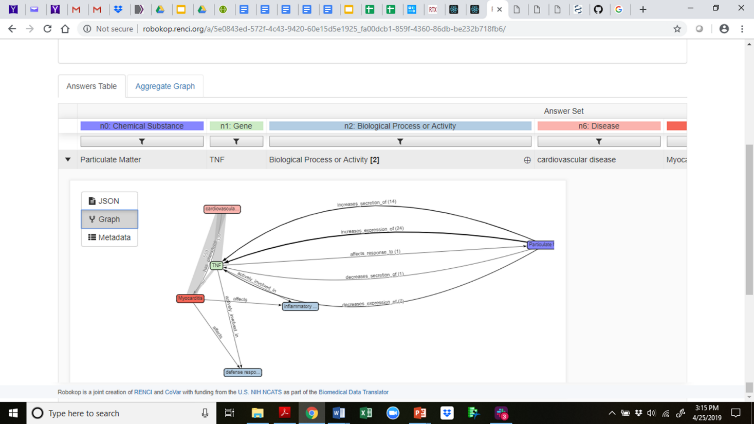
The top answer subgraph for this AOP is shown in Figure 6. In this example, ROBOKOP established a relationship between particulate matter and the *TNF* gene, with 47 total supporting PubMed publications and predicates that include “*increases secretion*”, “*increases expression*”, and “*affects response*”. In this answer subgraph, the supporting publications were derived from CTD.

**Fig. 6. Example answer subgraph for AOP query.**

The first citation for the relationship *particulate matter --> increases secretion of -->* TNF is shown in Figure 7.


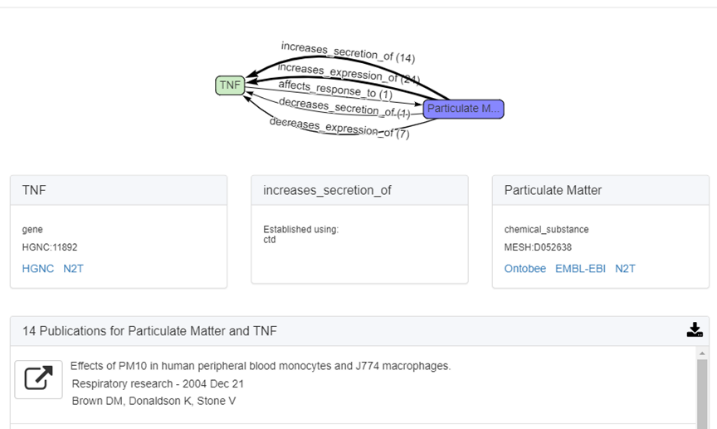


**Fig. 7. Example supporting evidence for AOP query answer.**
